# Cognitive–behavioural conjoint therapy versus prolonged exposure for PTSD in military service members and veterans: results and lessons from a randomized controlled trial

**DOI:** 10.1080/20008066.2024.2330305

**Published:** 2024-04-08

**Authors:** Candice M. Monson, Nicole D. Pukay-Martin, Anne C. Wagner, Alexander O. Crenshaw, Tabatha H. Blount, Richard P. Schobitz, Katherine A. Dondanville, Stacey Young-McCaughan, Jim Mintz, David S. Riggs, Antoinette Brundige, Elizabeth A. Hembree, Brett T. Litz, John D. Roache, Jeffrey S. Yarvis, Alan L. Peterson, for the STRONG STAR Consortium

**Affiliations:** a Department of Psychology, Toronto Metropolitan University, Toronto, Canada; b Cincinnati VA Medical Center, Cincinnati, OH, USA; c Remedy, Toronto, Canada; d Kennesaw State University, Kennesaw, GA, USA; e Department of Psychiatry and Behavioral Sciences, University of Texas Health Science Center at San Antonio, San Antonio, TX, USA; f Hérbert School of Medicine, Uniformed Services University of the Health Sciences, and Center for Deployment Psychology, Bethesda, MD, USA; g Department of Psychiatry, University of Pennsylvania, Philadelphia, PA, USA; h Massachusetts Veterans Epidemiological Research and Information Center, VA Boston Healthcare System, Boston, MA, USA; i Department of Psychological and Brain Sciences, Boston University, Boston, MA, USA; j Department of Psychiatry, Boston University School of Medicine, Boston, MA, USA; k Research and Development Service, South Texas Veterans Health Care System, San Antonio, TX, USA; l Department of Behavioral Health, Carl R. Darnall Army Medical Center, Fort Hood, TX, USA; m Department of Psychology, University of Texas at San Antonio, San Antonio, TX, USA; n Department of Population Health Sciences, University of Texas Health Science Center at San Antonio, San Antonio, TX, USA

**Keywords:** Military, PTSD, treatment, prolonged exposure, cognitive–behavioural conjoint therapy, couple therapy, intimate relationships, Militar, TEPT, tratamiento, exposición prolongada, terapia conjunta cognitiva conductual, terapias de pareja, relaciones íntimas

## Abstract

**Background:**

Military personnel and veterans are at heightened risk for exposure to traumatic events and posttraumatic stress disorder (PTSD), as well as intimate relationship problems associated with PTSD.

**Objective:**

The purpose of this study was to evaluate the relative efficacy of CBCT and PE in improving intimate relationship functioning in active duty military personnel or veterans and their intimate partners; both conditions were hypothesized to significantly improve PTSD. Method: In this study, 32 military service members or veterans with PTSD and their intimate partners were randomized to receive either Cognitive–Behavioral Conjoint Therapy for PTSD (*n* = 15; CBCT; [Monson, C. M., & Fredman, S. J. (2012). Cognitive-behavioral conjoint therapy for posttraumatic stress disorder: Harnessing the healing power of relationships. Guilford]), a trauma-focused couple therapy, or Prolonged Exposure (*n* = 17; PE; [Foa, E. B., Hembree, E. A., Dancu, C. V., Peterson, A. L., Cigrang, J. A., & Riggs, D. S. (2008). Prolonged exposure treatment for combat-related stress disorders – provider’s treatment manual [unpublished]. Department of Psychiatry, University of Pennsylvania]), a front-line evidence-based individual treatment for PTSD.

**Results:**

There were significant challenges with recruitment and a significant difference in dropout from treatment for the two therapies (65% for PE; 27% for CBCT). Treatment dropout was differentially related to pre-treatment relationship functioning; those with below average relationship functioning had higher dropout in PE compared with CBCT, whereas those with above average relationship functioning did not show differential dropout. In general, CBCT led to relational improvements, but this was not consistently found in PE. Clinician- and self-reported PTSD symptoms improved with both treatments.

**Conclusions:**

This study is the first to test a couple or family therapy against a well-established, front-line recommended treatment for PTSD, with expected superiority of CBCT over PE on relationship outcomes. Lessons learned in trial design, including considerations of equipoise, and the effects of differential dropout on trial analyses are discussed. This trial provides further support for the efficacy of CBCT in the treatment of PTSD and enhancement of intimate relationships.

## HIGHLIGHTS

- Differential dropout from trial of couple versus individual therapy for PTSD.
- General pattern of improvements in relationship outcomes in couple therapy for PTSD.
- PTSD symptoms improved in the individual and couple therapy for PTSD.
- Lessons learned in trial design, including considerations of equipoise, and the effects of differential dropout by condition on trial analyses are discussed.

## Abstract (es)

**Antecedentes:** El personal militar y los veteranos están en mayor riesgo de exposición a eventos traumáticos y el trastorno de estrés postraumático (TEPT), así como también a problemas de relaciones íntimas asociadas con el TEPT.

**Objetivo:** El propósito de este estudio fue evaluar la eficacia relativa de la Terapia Conjunta Cognitiva-Conductual (CBCT, en su sigla en inglés) y Exposición Prolongada (PE, en su sigla en inglés) en mejorar el funcionamiento de la relación intima en el personal militar en servicio activo o los veteranos y sus parejas intimas; se hipotetizó que ambas condiciones mejorarían significativamente del TEPT.

**Método**: En este estudio, 32 miembros del servicio militar o veteranos con TEPT y sus parejas intimas fueron asignados de forma aleatoria para recibir ya sea CBCT para TEPT (*n* = 15; CBCT; Monson & Fredman, 2012), una terapia de pareja centrado en el trauma, o PE (*n* = 17; PE; Foa et al., 2008), un tratamiento individual basado en la evidencia de primera línea para el TEPT.

**Resultados:** Hubo desafíos significativos con el reclutamiento y una diferencia significativa en el abandono al tratamiento para las dos terapias (65% para PE; 27% para CBCT). El abandono del tratamiento se relacionó diferencialmente con el funcionamiento de la relación antes del tratamiento; aquellos que tenían un funcionamiento de la relación bajo el promedio tuvieron un abandono más alto en PE comparado con CBCT, mientras que aquellos con un funcionamiento sobre el promedio no mostraron abandono diferencial. En general, CBCT resultó en mejoras relacionales, pero esto no fue encontrado en PE. Los síntomas TEPT clínicos y de autoinforme mejoraron en ambos tratamientos.

**Conclusiones:** Este estudio es el primero en evaluar la terapia de parejas o familias contra un tratamiento recomendado de primera línea, bien establecido para el tratamiento del TEPT, con superioridad esperada del CBCT por sobre el PE en los resultados de las relaciones. Se discutieron las lecciones aprendidas en el diseño de ensayos, incluyendo consideraciones de equilibrio, y los efectos del abandono diferencial en los análisis de ensayos. Este ensayo provee mayor apoyo para la eficacia del CBCT en el tratamiento del TEPT y la mejora de las relaciones íntimas.

Military personnel and veterans are at heightened risk for trauma exposure and posttraumatic stress disorder (PTSD; Judkins et al., 2020). In addition, there are well-documented associations between PTSD and intimate relationship problems, with even stronger associations found in military and veteran samples than in samples exposed to other types of trauma (see Taft et al., 2011 for meta-analysis). Increased recognition of the relational problems associated with PTSD, as well as compromised functioning in individuals partnered with patients with PTSD (Lambert et al., 2012), has led to the development of couple-based interventions for the treatment of PTSD (see Monson et al., 2021 for review). One of these interventions, Cognitive–Behavioral Conjoint Therapy (CBCT; Monson & Fredman, 2012) for PTSD, has accumulating evidence supporting its efficacy in treating PTSD and comorbid mental health conditions as well as improving relationship satisfaction and partner mental health (Liebman et al., 2020 for review). In spite of the growing empirical support for CBCT, no study has directly compared CBCT or any other couple/family therapy to a well-established individual treatment for PTSD, such as Prolonged Exposure (PE; Foa et al., 2008). The efficacy of PE in improving PTSD and comorbid symptoms is well established, but its effect on intimate relationship functioning is not yet documented (Sijercic et al., 2022).

The purpose of the present study was to evaluate the relative efficacy of CBCT and PE in enhancing intimate relationship functioning in active duty military personnel or veterans and their intimate partners. Given its focus on enhancing relationship factors in addition to improving PTSD, CBCT was hypothesized to result in significantly greater improvements in relational variables, including relationship satisfaction, relationship problems, fear of intimacy, and partner accommodation (i.e. partner tendencies to behaviourally accommodate the symptoms of PTSD; Fredman et al., 2014). Both CBCT and PE were hypothesized to improve clinician-, self-, and partner-rated PTSD symptoms, but no differences between them were predicted.

## 1. Method

### 1.1. Participants

**Figure 1.**
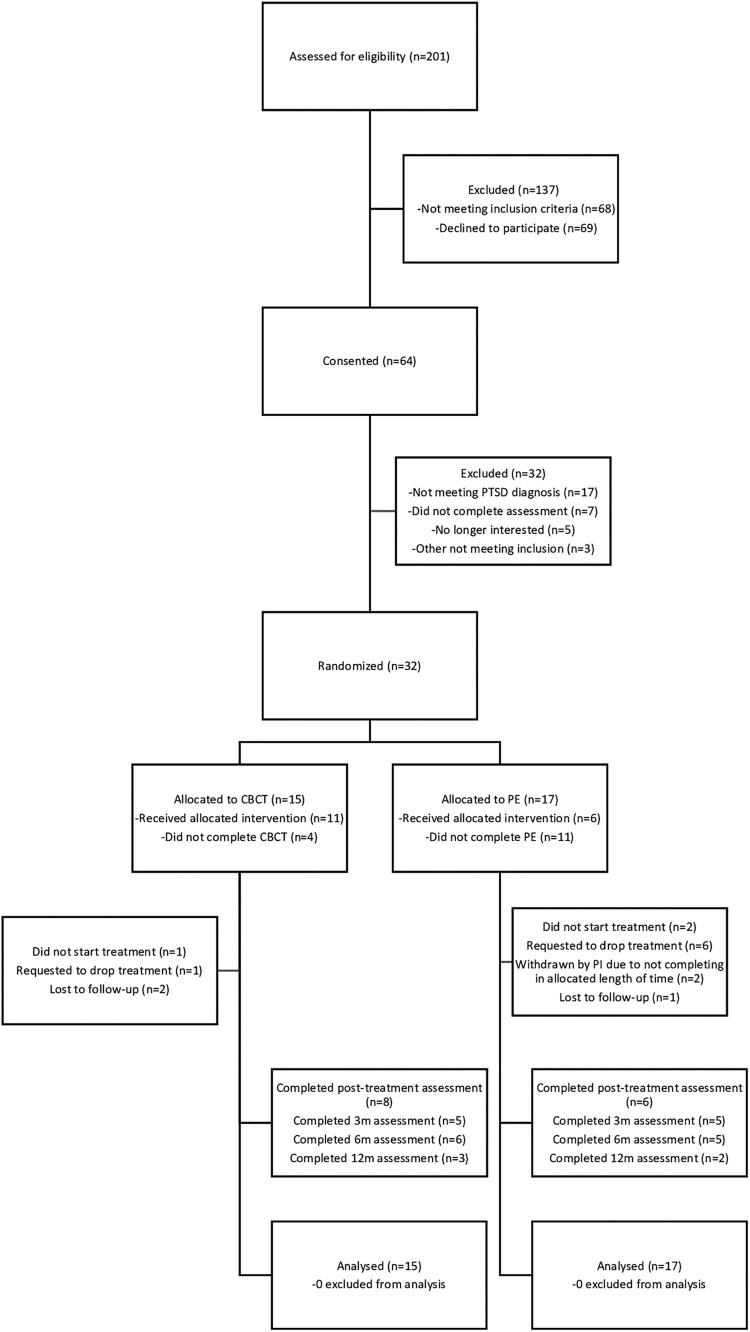
CONSORT flow diagram of eligibility, exclusion, randomization, and participation throughout the trial.

Participants were 32 couples consisting of US military personnel/veterans, and their intimate relationship partners who presented for treatment at Brooke Army Medical Center, Wilford Hall Medical Center, or the Carl R. Darnall Army Medical Center (see Table 1 for demographic and clinical characteristics). Inclusion criteria for the service member or veteran were at least one prior post-9/11 deployment to Iraq or Afghanistan and diagnosis of combat-related PTSD. Couples were required to be in an intimate relationship, either married or cohabitating, and cohabitating partners were required to be eligible for military health care through the Defense Enrollment Eligibility Reporting System. Additional eligibility criteria included that both partners were at least 18 years of age, English speaking, and, if taking psychotropic medications, on a stable medication regimen for 6 weeks. Exclusion criteria were severe intimate aggression within 6 months of study participation, current suicidal ideation warranting immediate attention, alcohol dependence, unmanaged psychotic or bipolar disorder, cognitive impairment that prohibited treatment, recent initiation of or an immediate need for other mental health treatment, and the partner meeting diagnostic criteria for PTSD. Details of recruitment and study participation are shown in the CONSORT diagram in Figure 1. The study was approved by the Institutional Review Boards at [redacted for review].

**Table 1.** Baseline characteristics by treatment condition and partner status.

Characteristic	PTSD-Identified Patients				Partners			
	CBCT (*n* = 15)	PE (*n* = 17)	*t,χ^2^*	*p*	CBCT (*n* = 15)	PE (*n* = 17)	*t,χ^2^*	*p*
Age	36.93 (6.32)	37.35 (6.51)	0.18	.855	36.13 (5.96)	37.71 (7.32)	0.66	.514
Male	14 (93.3%)	16 (94.1%)	0.01	.927	1 (6.7%)	1 (5.9%)	0.01	.927
Racial or Ethnic Minority	7 (46.7%)	7 (41.2%)	0.10	.755	7 (46.7%)	6 (35.3%)	0.43	.513
Married	15 (100%)	17 (100%)						
Length of Relationship	126.20 (89.34)	144.06 (87.77)	0.57	.573				
Total number of children	2.73 (1.75)	2.29 (1.21)	−0.83	.411				
Education			2.28	.516			6.30	.098
High school or less	1 (6.7%)	3 (17.6%)			1 (6.7%)	4 (23.5%)		
Some college/AA degree	10 (66.7%)	7 (41.2%)			7 (46.7%)	7 (41.2%)		
BA/BS degree	2 (13.3%)	3 (17.6%)			7 (46.7%)	3 (17.6%)		
Advanced degree	2 (13.3%)	4 (23.5%)			0 (0.0%)	3 (17.6%)		
Current Military Status			1.05	.306				
Active Duty	10 (66.7%)	14 (82.4%)						
Retired/Veteran	5 (33.3%)	3 (17.6%)						
Length Military Service	182.40 (87.91)	181.94 (82.21)	−0.02	.988				
Partner Served in Military					7 (46.7%)	7 (41.2%)	0.10	.755
Rank			2.05	.152				
Enlisted	13 (86.7%)	11 (64.7%)						
Officer	2 (13.3%)	6 (35.3%)						
Military Branch			3.15	.370				
Army	11 (73.3%)	16 (94.1%)						
Air Force	2 (13.3%)	1 (5.9%)						
Navy	1 (6.7%)	0 (0.0%)						
Marines	1 (6.7%)	0 (0.0%)						
Typical Duty			2.27	0.321				
Combat Arms	6 (40.0%)	8 (47.1%)						
Combat Support	7 (46.7%)	4 (23.5%)						
Combat Service Support	2 (13.3%)	5 (29.4%)						
Number of deployments			1.08	0.782				
1	5 (33.3%)	5 (29.4%)						
2	3 (20.0%)	6 (35.3%)						
3	3 (20.0%)	2 (11.8%)						
4+	4 (26.7%)	4 (23.5%)						

Note*.* CBCT = Cognitive-Behavioral Conjoint Therapy for Posttraumatic Stress Disorder. PE = Prolonged. Exposure. AA = Associate in Arts. BA = Bachelor of Arts. BS = Bachelor of Science.

### 1.2. Measures

The primary outcome measure of military member/veteran relationship satisfaction was assessed using the Couples Satisfaction Index (CSI; Funk & Rogge, 2007), a 32-item self-report measure. Items are summed to create a total score, with higher scores indicating greater relationship satisfaction (α** **= .98). Scores below a cut-score of 104.5 indicate relationally distressed status. Severity of problems within the relationship was measured by the 32-item Relationship Problems Scale (RPS; Riggs, 1993) with higher scores indicating greater relationship problem severity (α** **= .90). The Fear of Intimacy Scale (FIS; Descutner & Thelen, 1991) is a 35-item measure designed to assess an individual’s anxiety about close relationships with another person, with higher scores indicating greater anxiety (α = .93). Finally, partner accommodation behaviours in relation to service member or veteran PTSD symptoms were measured with the Significant Others’ Responses to Trauma Scale (SORTS; Fredman et al., 2014), which is a 14-item self-report measure of the frequency of distress related to accommodation behaviours. Items are summed, with higher scores indicating greater accommodation (α** **= .96).

PTSD clinician-rated symptoms and diagnostic status were assessed with the Clinician-Administered PTSD Scale for DSM-5 (CAPS-5; Weathers et al., 2013). The items were summed to create a total severity score, with higher scores indicating greater symptom severity (α** **= .94). The clinician assessors were trained and supervised within the South Texas Research Organizational Network Guiding Studies on Trauma and Resilience (STRONG STAR) Consortium (Peterson et al., 2021) and had excellent inter-rater reliability (Cohen’s kappa = 0.90). Service members and veterans completed the PTSD Checklist for DSM-5 (PCL-5; Weathers et al., 2013), a 20-item self-report measure assessing DSM-5 symptoms of PTSD, with higher scores indicating greater symptom severity (α** **= .93). Partners also completed the PCL-5 rating their perception of service member or veteran PTSD symptoms (α** **= .95; Ennis et al., 2021; Monson, 2012).

### 1.3. Procedure

Participants were recruited through flyers advertising for couples interested in a PTSD treatment study. These flyers were posted at Brooke Army Medical Center Wilford Hall Medical Center (subsequently renamed Wilford Hall Ambulatory Surgical Center), and the Carl R. Darnall Army Medical Center, clinician referral, relevant family support groups, and informational articles published in military papers. Interested individuals were provided information by phone or in person. After providing informed consent, service members, veterans, and their partners participated in baseline screening for eligibility. Couples were assigned with simple randomization to one of the two treatment conditions: CBCT or PE. Assessments were conducted at baseline, posttreatment, and 3 and 6 months after treatment completion.^1^ The CAPS-5 independent evaluators were blinded to treatment condition. All measures were administered at each assessment session. In addition, service members and veterans completed the PCL-5 and CSI at odd numbered treatment sessions. Those who dropped out of therapy were asked to continue completing assessments consistent with intention-to-treat principles.

#### 1.3.1. Treatment

CBCT (Monson & Fredman, 2012) is a 15-session trauma-focused therapy for PTSD delivered in a couple therapy format. It consists of three phases: (1) psychoeducation about PTSD and conflict management skills, (2) communication skills training and behavioural exposures designed to overcome avoidance, and (3) making new meaning of the trauma and addressing cognitions that maintain PTSD and/or relationship conflict via a dyadic cognitive strategy designed to increase cognitive flexibility in each partner. Sessions were 75 min long and occurred approximately weekly.

PE (Foa et al., 2008) is a gold-standard individual, trauma-focused therapy designed to decrease PTSD and comorbid symptoms. The therapy consists of imaginal exposure to specific trauma memories and *in vivo* exposures designed to overcome avoidance of specific trauma reminders. It was chosen as the comparison treatment due to the large evidence base supporting its efficacy and effectiveness. PE was delivered in 12 90-minute sessions on a weekly basis to equate the total therapy time to that of CBCT for PTSD. To assist with recruitment of couples, partners participated in the second session of PE, which discusses common trauma reactions and provides a rationale for in vivo exposure. Partners did not participate in the creation of the in vivo hierarchy.

Each therapy was delivered according to their respective manual by master’s and doctoral-level therapists who were trained to fidelity in each treatment prior to beginning the study. The therapists provided both of the therapies to control for therapist effects. They received weekly consultations from experts in each of the therapies who were part of the STRONG STAR Consortium (Peterson et al., 2021) to maintain their fidelity to the treatments.

### 1.4. Analytic approach

The present study originally sought to enrol 76 couples based on a power analysis on the primary relational outcome of relationship satisfaction, a medium between group effect size difference of *d* = .50, 20% assessment attrition, two-tailed significance testing, and power set at .80. The most conservative estimate of power, based on the lowest-power group (PE or CBCT) and lowest estimate of the correlation among time points with 17 treatment completers, indicates the study had a minimum of .8 power to detect within-treatment effect sizes of *d* = 0.97 for clinician-rated PTSD symptoms, *d *= 0.57 for self-reported PTSD symptoms, and *d *= 0.66 for relationship satisfaction, as well as between-group effect sizes of *d *= 0.58 for clinician-rated PTSD symptoms, *d *= 0.42 for self-reported PTSD symptoms, and *d *= 0.33 for relationship satisfaction. See Supplementary materials for details on power analyses.

Results were analysed for both the intention-to-treat (ITT) and completer samples. However, the pattern of dropout resulted in an imbalance between conditions, which compromised the internal validity of the study and thereby compromised the interpretation of ITT results and between-treatment comparisons (e.g. Lachin, 2000). Thus, we focused on analysis of within-condition effects in the treatment-completer sample, with analyses of the ITT sample to evaluate consistency of the results. We also focused on within-condition effects versus between-group effects for this reason and also limited the power to detect between-group differences due to the small sample size.

Multilevel growth models estimated with restricted maximum likelihood and a Kenward-Roger correction for small samples (McNeish & Stapleton, 2016) in the R (R Core Team, 2018) package lme4 (Bates et al., 2015) were used. Two-level models, with time points nested within individuals, were used for all outcomes. For outcomes provided for both partners (relationship outcomes, self, and partner reports of service member/veteran PTSD symptoms), dual-intercept models were used so each partner’s fixed and random effects could be estimated separately while accounting for dependency between partners. Each outcome was regressed onto time, dummy-coded treatment (0 = PE, 1 = CBCT), and the time by treatment interaction. A piecewise model was used for time, with separate slopes during the treatment phase (0 = baseline, 1 = posttreatment or follow-up)^2^ and follow-up phase (0 = posttreatment or earlier, 1 = 3-month follow-up, 2 = 6-month follow-up). Random intercepts were used for all models, as well as random slopes of time during treatment and intercept-slope correlations for service member and veteran self-reported PTSD symptoms and their relationship satisfaction. Random slopes of time during follow-up were not included due to sample size limitations, and only random intercepts were included for other outcomes due to lack of multiple measurement occasions during treatment for these outcomes. Likelihood ratio tests comparing a linear to quadratic effect of time during treatment were not significant (*p*s > .23), so a linear trajectory of change was used for all outcomes. Standardized effect sizes were computed by dividing raw estimates by the standard deviation of the variable at baseline (Feingold, 2009), separately for each partner, with a Hedges’ *g* correction for small samples (Hedges, 1981). As a sensitivity analysis, models were rerun by including significant baseline and demographic predictors of missing data–simplified to baseline relationship satisfaction and education (bachelor’s degree vs not) to minimize loss of degrees of freedom–as covariates. This approach provides unbiased estimates for multilevel models under the missing at random assumption (Graham, 2009).

## 2. Results

### 2.1. Treatment dropout and associated analyses

Treatment dropout was defined as not completing all sessions of the relevant protocol (i.e. 15 sessions of CBCT; 12 sessions of PE). There was a significant differential dropout rate by treatment condition, with 27% (*n* = 4) of CBCT and 65% (*n* = 11) of PE dropping out of treatment, χ^2^(1) = 4.49, *p *= .034. Notably, 18% (*n* = 2) of those assigned to PE dropped out of treatment without attending any sessions, and another 18% dropped out after the first session (compared with 7% (*n* = 1) and 7% in CBCT, respectively). To understand potential causes of differential dropout across conditions, we tested whether a range of demographic, baseline outcomes, and clinical characteristics predicted dropout on their own or when interacting with treatment condition in a series of logistic regressions (see Supplementary materials). The only potential predictors (*p* < .1) were service member or veteran relationship variables (relationship satisfaction *p* = .046, fear of intimacy *p* = .031, relationship problems *p* = .056) and education (*p* = .058) that interacted with condition to predict dropout. Johnson-Neyman regions of significance tests (Bauer & Curran, 2005) revealed that couples in which the service member or veteran rated the relationship as worse functioning than the mean had significantly higher dropout rates in PE compared with CBCT, whereas those who rated the relationship above the mean did not differ in dropout across conditions. Regarding education, those with less than a bachelor’s degree were significantly more likely to drop out of PE compared with CBCT (*p* = .009), but those with a bachelor’s degree or higher did not show differential dropout by condition (*p* = .819).

**Figure 2.**
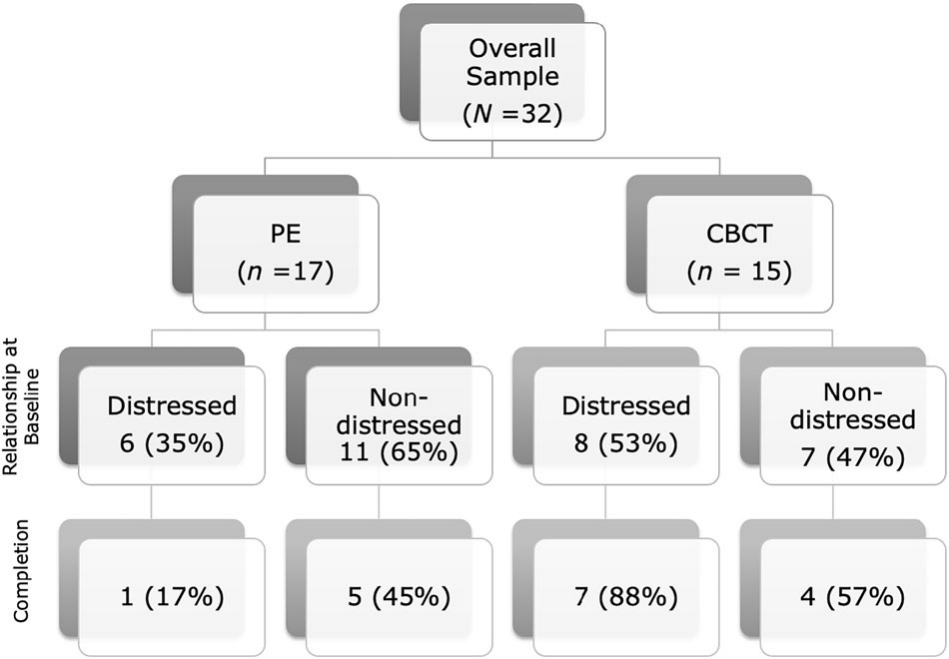
Rates of baseline relational distress and treatment dropout by condition.

To further illustrate dropout patterns by relationship functioning, Figure 2 shows dropout by condition and dichotomized relationship functioning (above vs below relational distress cutoff on satisfaction). 35% (*n* = 6) of the PE condition and 53% (*n* = 8) of the CBCT condition were relationally distressed at baseline. Approximately equal proportions of those who were relationally satisfied at pretreatment completed the two treatments. However, only 17% (*n* = 1) of relationally distressed participants randomized to PE completed treatment, whereas 88% (*n* = 7) of relationally distressed participants randomized to CBCT completed treatment.

### 2.2. Within-group change over treatment

**Figure 3.**
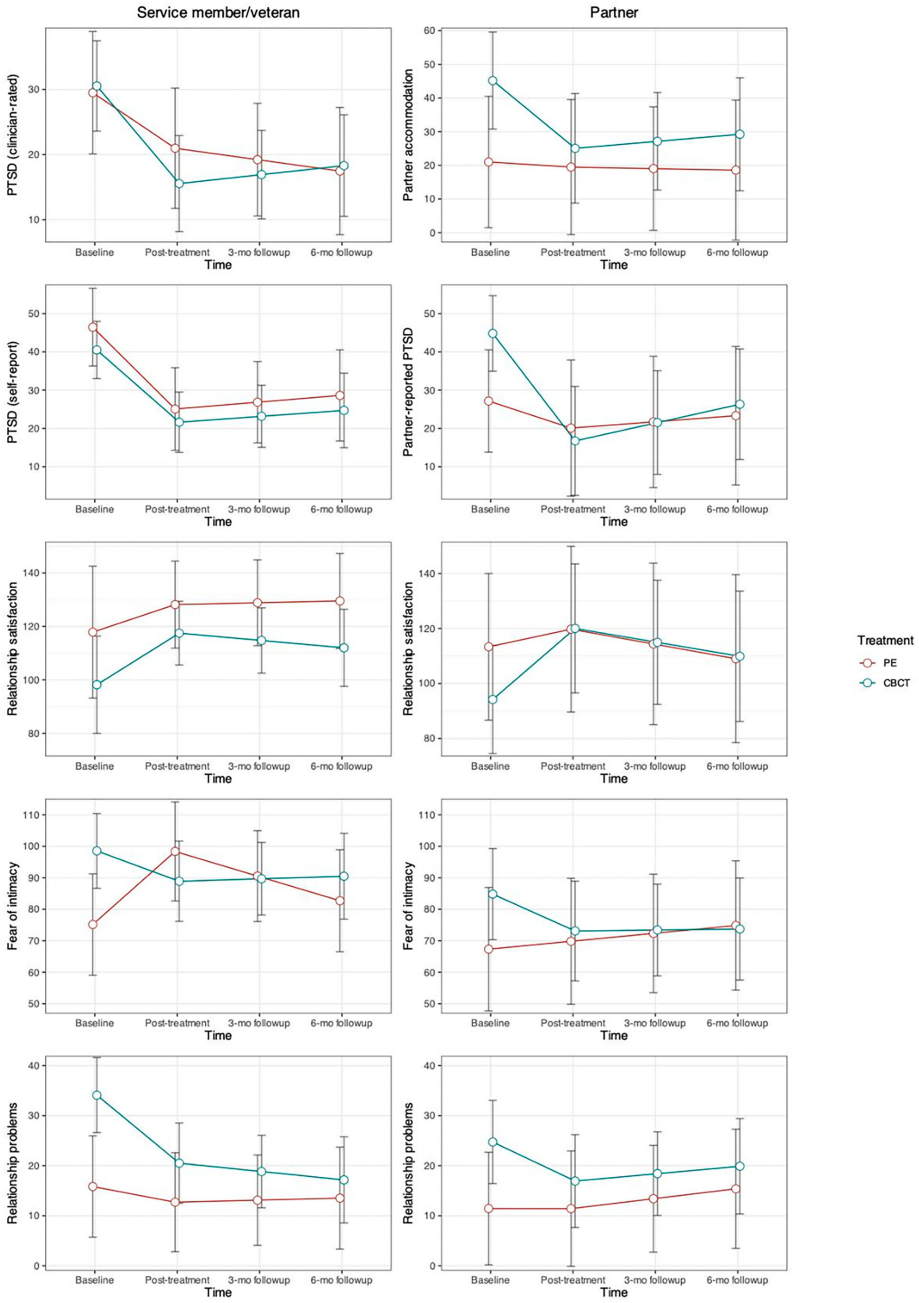
Estimated marginal means for each outcome by condition and partner.

Table 2 shows model-estimated marginal means and standardized effect size estimates for both partners and all outcomes in the completer sample, and Figure 3 shows plots of estimated marginal means for each outcome. For relationship outcomes, there was a general pattern in which CBCT resulted in significant improvements, whereas PE did not show evidence of improvement. This pattern was evident for service member/veteran relationship satisfaction, partners’ relationship satisfaction, and service member/veteran-rated relationship problems. Service member/veteran-rated fear of intimacy showed a significant worsening in PE. Similarly, partner accommodation was significantly improved in the CBCT condition, but not in PE. Results were consistent in the ITT sample (see Table 3), with a few exceptions. In addition to the other improvements in relationship measures, partners’ relationship problems were significantly improved in CBCT, and service member/veteran relationship satisfaction significantly improved in both conditions in the ITT sample. Service member/veteran fear of intimacy no longer showed significant worsening in PE for the ITT sample.

**Table 2.** Estimated marginal means and effect size estimates from multilevel models: completer sample.

Variable	Partner	Treatment	Estimated marginal means (CI)				Baseline to post-treatment *g* (CI)^a^	Baseline to 3m follow-up *g* (CI)^b^	Baseline to 6m follow-up *g* (CI)^b^
			Baseline	Post-treatment	3-month follow-up	6-month follow-up			
PTSD (clinician-rated)									
	Patient	PE	29.5 (20.1–38.9)	21.0 (11.7–30.2)	19.2 (10.6–27.9)	17.5 (7.7–27.2)	0.97 (0.08, 1.79)*	1.17 (0.43, 1.91)**	1.37 (0.43, 2.30)**
		CBCT	30.5 (23.6–37.5)	15.5 (8.2–22.9)	16.9 (10.1–23.7)	18.3 (10.5–26.1)	1.71 (1.01, 2.36)***	1.55 (0.93, 2.16)***	1.39 (0.62, 2.17)**
PTSD (self-/other- report)									
	Patient	PE	46.5 (36.3–56.6)	25.1 (14.3–35.9)	26.8 (16.2–37.5)	28.6 (16.7–40.5)	1.46 (0.82, 2.11)***	1.34 (0.70, 1.97)***	1.21 (0.50, 1.93)**
		CBCT	40.5 (33.0–48.0)	21.6 (13.7–29.5)	23.2 (15.1–31.3)	24.7 (15.0–34.4)	1.28 (0.80, 1.75)***	1.18 (0.69, 1.67)***	1.07 (0.47, 1.68)**
	Partner	PE	27.2 (13.8–40.5)	20.1 (2.3–37.9)	21.7 (4.5–38.9)	23.3 (5.2–41.4)	0.33 (−0.32, 1.03)	0.25 (−0.46, 0.96)	0.18 (−0.58, 0.93)
		CBCT	44.8 (34.9–54.7)	16.7 (2.5–31.0)	21.5 (8.0–35.1)	26.3 (11.9–40.8)	1.30 (0.73, 1.87)***	1.08 (0.51, 1.65)**	0.86 (0.24, 1.47)**
Partner accommodation									
	Partner	PE	21.0 (1.5–40.5)	19.5 (−0.6–39.5)	19.0 (0.7–37.4)	18.6 (−2.3–39.4)	0.05 (−0.49, 0.58)	0.06 (−0.40, 0.53)	0.07 (−0.50, 0.65)
		CBCT	45.2 (30.8–59.6)	25.0 (8.8–41.3)	27.1 (12.6–41.6)	29.2 (12.4–46.0)	0.62 (0.21, 1.04)**	0.56 (0.17, 0.94)**	0.49 (0.02, 0.97)*
Relationship satisfaction									
	Patient	PE	118 (93–143)	128 (112–144)	129 (113–145)	130 (112–147)	0.26 (−0.16, 0.72)	0.28 (−0.16, 0.71)	0.29 (−0.18, 0.76)
		CBCT	98 (80–116)	118 (106–129)	115 (103–127)	112 (98–126)	0.48 (0.18, 0.83)**	0.42 (0.09, 0.75)*	0.35 (−0.03, 0.72)
	Partner	PE	113.3 (87–140)	120 (90–150)	114 (85–144)	109 (79–140)	0.16 (−0.34, 0.68)	0.03 (−0.49, 0.55)	−0.11 (−0.67, 0.45)
		CBCT	94.2 (75–114)	120 (97–144)	115 (92–138)	110 (86–134)	0.65 (0.24, 1.07)**	0.53 (0.11, 0.94)*	0.40 (−0.06, 0.85)
Fear of intimacy									
	Patient	PE	75.2 (59.1–91.3)	98.4 (82.6–114.1)	90.5 (76.1–105.0)	82.7 (66.5–98.9)	−1.00 (−1.56, −0.44)**	−0.66 (−1.17, −0.15)*	−0.32 (−0.94, 0.29)
		CBCT	98.5 (86.7–110.4)	88.9 (76.2–101.6)	89.7 (78.2–101.2)	90.5 (76.9–104.1)	0.41 (−0.08, 0.91)	0.38 (−0.05, 0.81)	0.35 (−0.19, 0.88)
	Partner	PE	67.3 (47.7–86.9)	69.8 (49.8–89.8)	72.3 (53.5–91.2)	74.8 (54.3–95.4)	−0.11 (−0.73, 0.54)	−0.22 (−0.79, 0.34)	−0.33 (−1.03, 0.36)
		CBCT	84.8 (70.3–99.3)	73.1 (57.3–89.0)	73.4 (58.9–88.0)	73.7 (57.5–89.9)	0.52 (0.01, 1.03)^c^	0.51 (0.04, 0.97)*	0.49 (−0.09, 1.07)
Relationship problems									
	Patient	PE	15.8 (5.7–26.0)	12.7 (2.8–22.6)	13.1 (4.1–22.1)	13.5 (3.3–23.7)	0.18 (−0.29, 0.64)	0.15 (−0.28, 0.59)	0.13 (−0.39, 0.65)
		CBCT	34.1 (26.6–41.6)	20.5 (12.5–28.5)	18.8 (11.6–26.1)	17.2 (8.5–25.8)	0.77 (0.36, 1.18)***	0.87 (0.50, 1.23)***	0.96 (0.50, 1.42)***
	Partner	PE	11.4 (0.2–22.7)	11.4 (−0.1–22.9)	13.4 (2.7–24.1)	15.4 (3.5–27.3)	0.00 (−0.56, 0.62)	−0.12 (−0.64, 0.39)	−0.25 (−0.88, 0.38)
		CBCT	24.7 (16.4–33.0)	16.9 (7.7–26.2)	18.4 (10.1–26.8)	19.9 (10.4–29.4)	0.49 (0.01, 1.00)^c^	0.40 (−0.03, 0.83)	0.31 (−0.22, 0.83)

Note. Positive effect sizes represent improvement. CI = 95% confidence intervals. Effect sizes are the estimated change from multilevel models divided by the standard deviation of the outcome variable at baseline, with a Hedge’s *g* correction due to effect size inflation at small samples (Hedges, 1981).

^a^ Confidence intervals obtained using parametric bootstrapping with 1000 resamples.

^b^ Confidence intervals obtained from planned contrasts via estimated marginal means.

^c^ Wald test statistic was not significant despite bootstrapped CIs excluding 0, as these values are obtained via separate means.

* *p* < .05. ** *p* < .01. *** *p* < .001.

**Table 3.** Estimated marginal means and effect size estimates from multilevel models: intent-to-treat sample.

Variable	Partner	Treatment	Estimated marginal means (CI)				Baseline to post-treatment *g* (CI)^a^	Baseline to 3m follow-up *g* (CI)^b^	Baseline to 6m follow-up *g* (CI)^b^
			Baseline	Post-treatment	3-month follow-up	6-month follow-up			
PTSD (clinician-rated)									
	Patient	PE	32.8 (27.8–37.9)	25.5 (18.7–32.4)	22.8 (16.7–28.9)	20.1 (12.1–28.1)	0.83 (0.11, 1.56)*	1.14 (0.49, 1.79)**	1.45 (0.57, 2.33)**
		CBCT	30.3 (24.9–35.7)	15.6 (9.2–22.1)	17.3 (11.7–22.9)	19.0 (12.4–25.7)	1.66 (0.96, 2.30)***	1.47 (0.90, 2.04)***	1.28 (0.56, 1.99)**
PTSD (self-/partner- report)									
	Patient	PE	46.7 (41.0–52.4)	29.7 (20.3–39.0)	30.7 (21.4–40.1)	31.8 (20.8–42.8)	1.16 (0.56, 1.79)**	1.09 (0.44, 1.74)**	1.02 (0.27, 1.77)**
		CBCT	42.8 (36.9–48.6)	22.4 (14.9–29.9)	24.3 (16.6–32.0)	26.2 (17.0–35.5)	1.39 (0.94, 1.89)***	1.26 (0.73, 1.78)***	1.13 (0.51, 1.74)**
	Partner	PE	37.7 (28.5–46.8)	28.2 (11.0–45.5)	29.8 (13.3–46.4)	31.4 (13.9–49.0)	0.44 (−0.28, 1.12)	0.36 (−0.35, 1.08)	0.29 (−0.47, 1.05)
		CBCT	45.2 (35.5–54.9)	18.2 (3.7–32.7)	23.4 (9.8–37.0)	28.6 (14.3–42.9)	1.25 (0.65, 1.80)***	1.01 (0.46, 1.56)**	0.77 (0.19, 1.35)*
Partner accommodation									
	Partner	PE	33.2 (20.3–46.1)	26.5 (6.4–46.6)	26.1 (8.9–43.2)	25.7 (4.4–47.0)	0.21 (−0.40, 0.80)	0.22 (−0.29, 0.73)	0.23 (−0.41, 0.88)
		CBCT	49.4 (35.7–63.1)	29.5 (11.8–47.3)	34.9 (20.1–49.7)	40.2 (22.7–57.7)	0.61 (0.08, 1.12)*	0.45 (0.02, 0.88)*	0.28 (−0.24, 0.81)
Relationship satisfaction									
	Patient	PE	102.4 (86–119)	119.8 (107–133)	120.6 (108–133)	121.3 (106–136)	0.44 (0.09, 0.78)*	0.46 (0.12, 0.79)**	0.48 (0.09, 0.86)*
		CBCT	105.0 (87–123)	121.0 (110–133)	118.4 (107–130)	115.7 (102–129)	0.40 (0.13, 0.70)*	0.34 (0.03, 0.64)*	0.27 (−0.08, 0.61)
	Partner	PE	96.5 (79–114)	104.9 (76–134)	100 (72–127)	94 (65–123)	0.21 (−0.36, 0.81)	0.08 (−0.54, 0.69)	−0.06 (−0.70, 0.59)
		CBCT	93.5 (75–112)	113.5 (89–138)	108 (84–132)	103 (78–127)	0.51 (0.08, 0.94)*	0.37 (−0.10, 0.83)	0.23 (−0.26, 0.72)
Fear of intimacy									
	Patient	PE	90.5 (79.6–101.3)	99.8 (85.6–114.1)	94.9 (82.1–107.6)	89.9 (74.3–105.5)	−0.40 (−0.95, 0.14)	−0.19 (−0.68, 0.30)	0.02 (−0.60, 0.65)
		CBCT	94.5 (82.9–106.0)	86.3 (72.7–99.9)	87.2 (75.3–99.1)	88.2 (74.2–102.1)	0.35 (−0.15, 0.88)	0.31 (−0.12, 0.75)	0.27 (−0.27, 0.81)
	Partner	PE	72.5 (61.7–83.2)	73.0 (57.0–89.1)	75.7 (61.8–89.6)	78.4 (61.5–95.3)	−0.02 (−0.71, 0.65)	−0.14 (−0.71, 0.42)	−0.26 (−0.98, 0.45)
		CBCT	85.6 (74.2–97.0)	73.8 (59.4–88.1)	74.9 (62.7–87.2)	76.1 (62.0–90.3)	0.53 (−0.06, 1.10)	0.47 (0.00, 0.94)*	0.42 (−0.15, 1.00)
Relationship problems									
	Patient	PE	23.7 (17.2–30.2)	17.8 (9.3–26.4)	18.2 (10.5–25.9)	18.6 (9.2–28.0)	0.33 (−0.11, 0.76)	0.31 (−0.07, 0.70)	0.29 (−0.21, 0.79)
		CBCT	31.2 (24.2–38.2)	18.9 (10.7–27.0)	17.5 (10.3–24.7)	16.2 (7.8–24.6)	0.70 (0.31, 1.09)**	0.78 (0.43, 1.12)***	0.85 (0.42, 1.28)***
	Partner	PE	19.3 (12.4–26.2)	16.7 (6.8–26.7)	18.7 (10.0–27.4)	20.7 (10.2–31.2)	0.16 (−0.40, 0.73)	0.04 (−0.45, 0.53)	−0.09 (−0.70, 0.53)
		CBCT	26.8 (19.5–34.1)	18.8 (9.8–27.8)	20.9 (13.1–28.6)	22.9 (14.1–31.8)	0.51 (0.03, 0.99)*	0.38 (−0.03, 0.78)	0.24 (−0.25, 0.74)

Note. Positive effect sizes represent improvement. CI = 95% confidence intervals. Effect sizes are the estimated change from multilevel models divided by the standard deviation of the outcome variable at baseline, with a Hedge’s *g* correction due to effect size inflation at small samples (Hedges, 1981).

^a^ Confidence intervals obtained using parametric bootstrapping with 1000 resamples.

^b^ Confidence intervals obtained from planned contrasts via estimated marginal means.

* *p* < .05. ** *p* < .01. *** *p* < .001.

For PTSD outcomes, there were significant and large effect size improvements in clinician-rated and self-reported PTSD symptoms in both conditions. Partner-reported PTSD symptoms were significantly improved in the CBCT condition, but not in PE. Results were consistent in the ITT sample (see Table 3). Across outcomes, gains were generally maintained at follow-up. Except for loss of significance in clinician-rated PTSD in PE for ITT models, magnitude, direction, and significance of results were unchanged when controlling for predictors of missing data.

### 2.3. Between-group differences in change

CBCT resulted in significantly better outcomes for partner-reported PTSD symptoms and service member/veteran intimacy in the completer sample; other between-group differences were nonsignificant. All between-group differences were nonsignificant in the ITT sample, although there were some large effect size differences (see Table 4). Except for the service member/veteran’s fear of intimacy, which had a significant group difference favouring CBCT in ITT models, results were unchanged when controlling for predictors of missing data.

**Table 4.** Between-group differences in treatment efficacy.

Variable	Service Member/Veteran or Partner	*Completer sample* Pre-Post Change Difference *g* (CI)		*ITT sample* Pre-Post Change Difference *g* (CI)

PTSD (clinician-rated)	Service Member/Veteran	0.74 (−0.36, 1.85)		0.84 (−0.14, 1.84)
PTSD (self-report)	Service Member/Veteran	−0.17 (−0.97, 0.62)		0.23 (−0.58, 1.03)
PTSD (partner report)	Partner	0.97 (0.08, 1.91)*		0.81 (−0.01, 1.66)
Partner accommodation	Partner	0.57 (−0.05, 1.28)		0.40 (−0.41, 1.17)
Relationship satisfaction	Service Member/Veteran	0.23 (−0.33, 0.75)		−0.04 (−0.47, 0.42)
	Partner	0.49 (−0.17, 1.12)		0.30 (−0.48, 0.97)
Fear of intimacy	Service Member/Veteran	1.41 (0.65, 2.16)*		0.75 (−0.02, 1.53)
	Partner	0.63 (−0.24, 1.52)		0.55 (−0.36, 1.47)
Relationship problems	Service Member/Veteran	0.59 (−0.00, 1.27)		0.37 (−0.22, 0.96)
	Partner	0.49 (−0.30, 1.27)		0.34 (−0.41, 1.13)

Note. CI = confidence interval, Positive effect sizes represent greater improvement in CBCT condition, whereas negative effect sizes represent greater improvement in PE condition. CI = 95% confidence intervals from parametric bootstrapping with 1000 resamples. Effect sizes are the estimated change from multilevel models divided by the standard deviation of the outcome variable at baseline, with a Hedge’s *g* correction due to effect size inflation at small samples (Hedges, 1981).

* *p* < .05.

## 3. Discussion

This study is the first to test a couple or family therapy against a well-established, front-line recommended treatment for PTSD, with expected superiority of CBCT over PE on relationship outcomes. Improvements in PTSD in both conditions were expected, with no differences between them. As expected, between-group differences were largely non-significant, but this should be cautiously interpreted due to issues with power and imbalance in the two conditions due to a higher dropout rate in the PE group. Nonetheless, these results do provide additional evidence for the efficacy of CBCT for PTSD to improve relationship outcomes in patients with PTSD and replicate the well-established effectiveness of PE for PTSD – at least for those with sufficiently strong relationships so as to stay in treatment.

In addition to providing additional outcome data, this trial offers a series of lessons to the field in the design and analyses of randomized clinical trials. These lessons include the intricacies of recruiting for randomization to different therapy formats and the related principle of equipoise, differential dropout and ‘undoing’ randomization, and ITT versus completer analyses when there is differential dropout. Equipoise, or the genuine uncertainty on the part of the clinical investigator about the relative effects of each treatment arm, is considered an essential ingredient of randomized clinical trials (Freedman, 1987). Sentinel findings of this study were the significantly different treatment dropout rates between the two conditions and difficulties in recruitment. The likelihood of dropping out of PE was over two times greater than from CBCT (65% versus 27%). As a point of comparison, the average dropout rate from a 10-session weekly PE protocol tested in another study in our STRONG STAR research consortium was 24.8% (Foa et al., 2018). The disparate dropout rate, and the fact that nearly half the dropout from PE occurred prior to the initiation of exposure exercises, leads us to believe that military personnel and veterans who sought this treatment study involving intimate partners correspondingly had a preference to be randomized to the CBCT treatment because it was a couples’ therapy and explicitly focused on relationship functioning. In contrast, although the partners were invited to attend one PE treatment session (session 2), PE is considered an individual treatment.

The presumed association between an interest in relationship functioning and treatment preference is important in and of itself. It is important to acknowledge that the population of PTSD sufferers is heterogeneous and their reasons for seeking treatment and the treatment(s) they prefer are likewise varied. Prior studies document that close relationships are significant facilitators of PTSD treatment seeking (Spoont et al., 2014), and couple/family relationship functioning is associated with likelihood of staying in trauma-focused PTSD treatment (Meis et al., 2019) and outcomes achieved (Tarrier et al., 1999). Additionally, the importance of patient preference in treatment engagement and outcomes for a variety of conditions is well documented (Windle et al., 2020 for meta-analysis). Thus, it is important to offer a range of effective treatments for those who suffer from PTSD and their loved ones in order to maximize the likelihood that they will engage in, and profit from, treatment.

The differential dropout rate in this study points to patient and partner preference or the lack of personal equipoise for those who came to the study, but it also raises questions about equipoise more broadly in the trial design. Although intuitively appealing to conduct an individual versus couple/family treatment for a range of conditions, we had not considered *a priori* that participants might not be equally poised to receive both treatment arms. We believe that this contributed to significant difficulties in recruiting for the study in a consortium that had significant success in recruiting for a number of treatment trials (Peterson et al., 2021).

For those who chose to participate in the trial, a major methodological and statistical consequence was that randomization was ‘undone’ with unequal treatment dropout rates. The predictors of dropout buttress the argument that relationship factors were salient to those who came to the trial, given that those with poorer baseline relationship functioning were more likely to dropout from PE. Moreover, the finding that those with lower education were more likely to dropout from PE suggests that there may be a larger constellation of psychosocial concerns that place a patient at risk for dropout from an individual therapy for PTSD. We hope that our cautionary tale might fuel future adaptive designs and preference trials that may mitigate these internal and external validity issues.

Additional limitations to the trial include small sample size, which may have yielded less stable estimates of the treatment effects. In spite of the sample size, significant within-treatment effects were found due to the large treatment effects. The sample was also limited in terms of its diversity. The majority of service members and veterans were White, non-Hispanic, males with female partners and all were married. Future studies should seek to engage a more inclusive, heterogeneous sample in terms of race, ethnicity, gender, and composition of dyads. Longer-term follow-up to better examine maintenance of gains would also be useful in future studies.

There are a number of individual treatments for PTSD that are efficacious and well-tolerated by patients. Yet, there is a substantial number of individuals who do not respond or only partially respond to these treatments. Given that intimate-relationship problems often co-occur with PTSD, it may complicate trauma recovery and is a reason that those with PTSD seek treatment. Efforts to develop efficacious couple/family therapies that can simultaneously improve PTSD and relationship problems are warranted. This trial provides further support for the efficacy of CBCT in the treatment of PTSD and enhancement of intimate relationships.

Notes1An assessment at 12-months posttreatment was also included, but only four couples completed this assessment, so this time point was excluded from analyses.2To create the time variable during treatment, the session number was divided by the total number of sessions for the therapy so that 0 = baseline and 1 = posttreatment in both conditions.

## Supplementary Material

Supplemental materials.docx

## Data Availability

The data from this study are maintained at the University of Texas Health Science Center at San Antonio in the STRONG STAR Repository. Requests for access to the data as well as for materials and the analysis code also can be emailed to repository@strongstar.org.
