# Supplementary material for: Cognitive–behavioural conjoint therapy versus prolonged exposure for PTSD in military service members and veterans: results and lessons from a randomized controlled trial
Source: Eur J Psychotraumatol. 2024 Apr 8;15(1):2330305. doi: 10.1080/20008066.2024.2330305 (PMC11005874; doi:10.1080/20008066.2024.2330305)
Supplement: Supplemental materials.docx [file ZEPT_A_2330305_SM0577.docx]

Supplemental Materials

**Additional details on Johnson-Neyman regions of significance tests for missing data analysis**

To understand potential causes of differential dropout across conditions, we tested whether the following variables predicted dropout on their own or when interacting with treatment condition (testing differential associations across conditions) in a series of logistic regressions: baseline values of all outcome variables for both partners, and MPV’s age, relationship length, dichotomized education (below vs at least a bachelor’s degree), dichotomized race (White vs other categories), and self-reported current depression. No baseline values of outcome variables significantly predicted dropout on their own, and neither PTSD outcomes nor the partner’s relationship outcomes significantly interacted with condition to predict dropout. However, the combined evidence indicates differential rates of dropout by condition depending on the MPV’s relationship variables (variable by condition interaction: relationship satisfaction *p* = .046, fear of intimacy *p* = .031, relationship problems *p* = .056).

Johnson-Neyman regions of significance tests (Bauer & Curren, 2005) found a general pattern in which couples for whom the MPV rated the relationship as worse functioning than the mean had significantly higher dropout rates in PE compared with CBCT, whereas those who rated the relationship above the mean did not differ in dropout across conditions. However, the specific cut point in the baseline relationship variable at which there was differential dropout by condition differed slightly for the three relationship variables (see Figure A1). This cut point--where differential dropout by condition began to emerge--was below +0.18 *SD* of the mean for relationship satisfaction (where lower = worse functioning), above +0.24 *SD* of the mean for fear of intimacy (where higher = worse functioning), and above +0.02 *SD* of the mean for relationship problems (where higher = worse functioning). Of demographic variables tested, there was a marginal interaction between education and condition in predicting dropout (*p* = .058), in which those with less than a bachelor’s degree were significantly more likely to drop out in PE compared with CBCT (*p* = .009), but those with a bachelor’s degree or higher did not show differential dropout by condition. No other demographic variables predicted dropout on their own or in interaction with condition.

**Figure A1**

*Johnson-Neyman regions of significance plots for relationship outcomes*

**
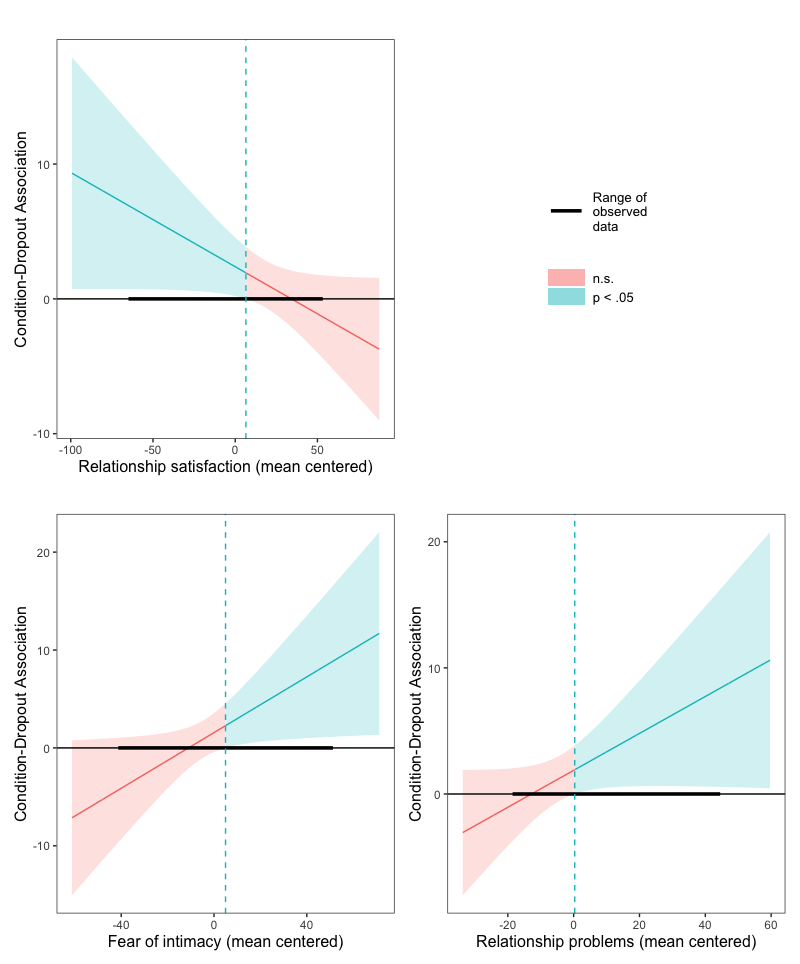
**
